# Supplementary material for: Simple mechanistic traits outperform complex syndromes in predicting avian dispersal distances
Source: Commun Biol. 2026 Feb 11;9:376. doi: 10.1038/s42003-026-09676-x (PMC12992614; doi:10.1038/s42003-026-09676-x)
Supplement: Supplementary file 1 — Supplementary Information [file 42003_2026_9676_MOESM1_ESM.docx]

**Supporting Information**

Simple mechanistic traits outperform complex syndromes in predicting avian dispersal distances

Guillermo Fandos, Robert A. Robinson, Damaris Zurell

Corresponding author: Guillermo Fandos.

**Email:**  gfandos@ucm.es

**Supplementary Information:**

Table of contents

[Supplementary Note 1: Trait data imputation 3](#_Toc220334843)

[Supplementary Note 2: Alternative phylogenetic comparative analysis using PGLS 5](#_Toc220334844)

[Supplementary Note 3: Phylogenetic generalized linear mixed models without interactions 7](#_Toc220334845)

[Supplementary Note 4: Interaction models 9](#_Toc220334846)

[Supplementary Note 5: Sensitivity analyses 13](#_Toc220334847)

[Supplementary Tables 17](#_Toc220334848)

[Supplementary References 28](#_Toc220334849)

## **Supplementary Note 1: Trait data imputation**

Because trait data (Supplementary Table 1) were not available for all species in our data set, we used phylogenetic trait imputation to fill gaps in our data set. Trait imputation can provide reliable information for up to 60% of missing data, and adding phylogenetic information to trait imputation has been shown to strongly reduce estimation error ^1^. However, estimation error may be higher when closely related species have missing data. We used two imputation methods available. i) a random forest imputation algorithm in combination with phylogenetic information with the missForest function in the R package missForest ^2^. For each trait, we assessed imputation error for a range of phylogenetic eigenvectors (1–30) and used the imputed trait values for the number of phylogenetic eigenvectors that minimized imputation error. ii) Mice, multiple imputation chained equations^3^.

Our gap-filled trait data had similar distributions and median values as the observed trait data (Supplementary Figure 1). However, results were sensitive to the imputed trait data, especially when we combine trait imputation with phylogenetic information. Thus, we decided to remove species with missing values from the analysis, yet also present results excluding species with imputed trait data. The complete datasets with all traits were 138 species for total dispersal, 63 for breeding dispersal and 72 species for natal dispersal.

Furthermore, we developed an analysis to explore the relationship between species traits and dispersal distances while accounting for phylogenetic relatedness and missing data; we implemented a Bayesian phylogenetic mixed model using the brms package in R^4^. Our approach involved multiple imputations to handle missing values and incorporated a phylogenetic covariance matrix to model species-level correlations. We fitted a Bayesian phylogenetic mixed model using brm_multiple() from the brms package, which allows multiple imputed datasets to be analyzed within a unified Bayesian framework. The use of multiple imputations ensured that uncertainty in missing values was propagated into the final model estimates. Supplementary Figure 1 show that some variables increase the importance of explaining median and long-distance dispersal compared to results for the dataset without data imputation (n= 138 species; Fig. 1), such as migration distance for median dispersal or life-history for long-distance dispersal.


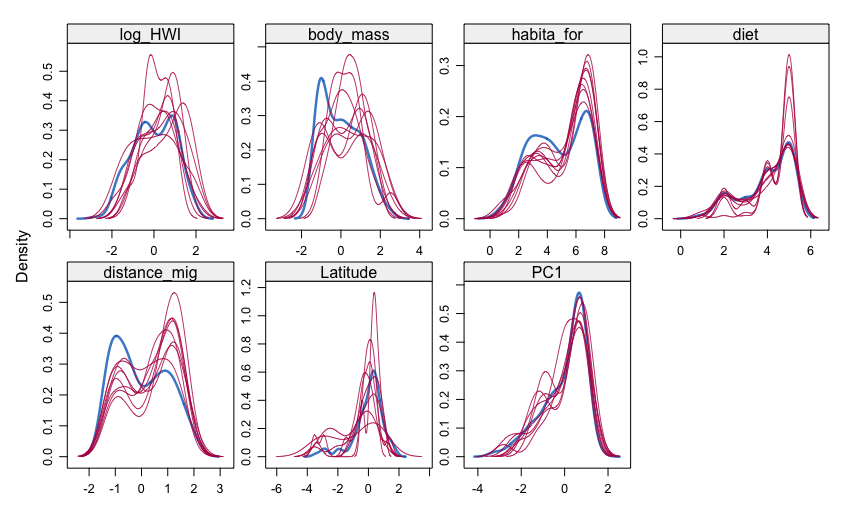


Supplementary Figure 1: Comparison of observed and imputed trait distributions. Density plots showing the distribution of observed (orange) and imputed (teal) trait values using the MICE (Multivariate Imputation by Chained Equations) approach. Traits shown: (a) Hand-Wing Index (log-transformed), (b) body mass (log-transformed), (c) habitat openness (forest–open gradient), (d) diet (herbivore–carnivore gradient), (e) migration distance, (f) breeding latitude, and (g) life-history pace (PC1, slow–fast continuum). Observed traits: n = 138 species; imputed traits: n = 234 species.


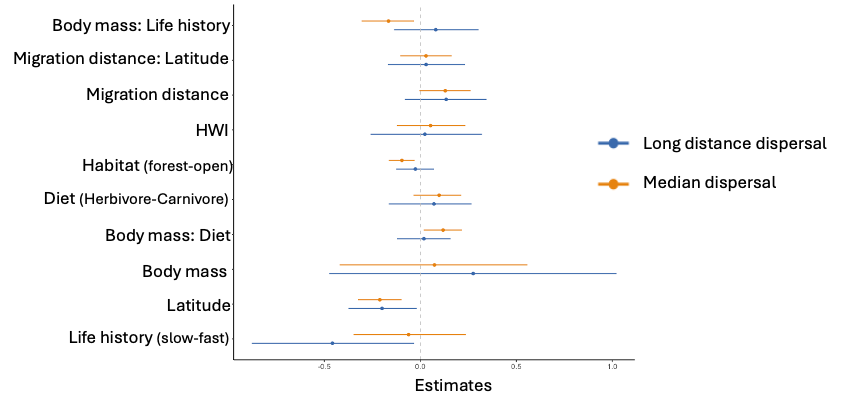


Supplementary Figure 2. Dispersal syndromes using imputed trait data. Standardized coefficients and 95% credible intervals of predictors of median (orange circles) and long-distance (blue circles) total dispersal distances among European birds (n = 234 species) based on phylogenetic generalized linear mixed models with imputed trait data. Individual data points represent species-level residuals. Dispersal estimates stem from the Weibull distribution ^5^. Error bars represent 95% credible intervals from posterior distributions.

## **Supplementary Note 2: Alternative phylogenetic comparative analysis using PGLS**

**Justification**

In the manuscript, we used a Bayesian modeling approach implemented in the brms package ^4^, incorporating phylogenetic structure via a covariance matrix. We performed model selection using the projection predictive variable selection (projpred; ^6^), which allows for a principled reduction of model complexity while retaining predictive accuracy. This approach provides a flexible and probabilistic framework for assessing predictor effects.

To ensure the robustness of our results and compare and validate our findings, in this supporting material we applied a frequentist approach using phylogenetic generalized least-squares (PGLS) models ^7^ with model selection and model averaging ^8^. The consistency of key predictors across both methods reinforces the reliability of our results.

**Methods**

We assessed the relationship between predictors and natal dispersal distance using phylogenetic generalized least-squares (PGLS) models ^7^ in combination with model selection and model averaging approaches ^8^, implemented in R 4.4.1. To account for phylogenetic non-independence among species, we incorporated a covariance structure where the parameter λ controls the strength of the phylogenetic signal.

Our analyses were based on a phylogenetic tree including 138 species, constructed from a sample of phylogenetic trees. Total dispersal values were log-transformed to improve homoscedasticity. Similarly, Hand Wing Index (HWI), and body mass were log-transformed to enhance model fit and residual normality.

Then we used information-theoretic model selection and multimodel inference techniques for identifying optimal models that balance fit and complexity ^8^. We calculated the Akaike information criterion (AICc), relative model likelihoods, and model probabilities based on differences in AICc values (ΔΑΙCc).

We used the MuMIn package in R ^9^ to perform model selection and develop a model total based on the top models comprising 95% of the cumulative AIC weight. First, we fitted a global model and generated a set of candidate models using the dredge() function, which systematically evaluates all possible subsets of predictors. We then identified the models contributing to 95% of the cumulative Akaike weight using the subset argument in get.models(). Finally, we applied the model.avg() function to calculate a weighted total of the model parameters, accounting for model uncertainty. This approach provides robust parameter estimates while correcting for phylogenetic non-independence and allows us to assess the relative importance of predictors.

**Results**

The results from both approaches were highly consistent, reinforcing the reliability of our findings. In this case, body mass and life history emerged as the most important predictors of dispersal distance, being the only variables with an importance score exceeding 0.8 (Supplementary Figure 3).

Body mass, life history, their interaction, and latitude were the strongest predictors of total dispersal distance, with significant effects in both the Bayesian and PGLS models (Supplementary Figure 3).


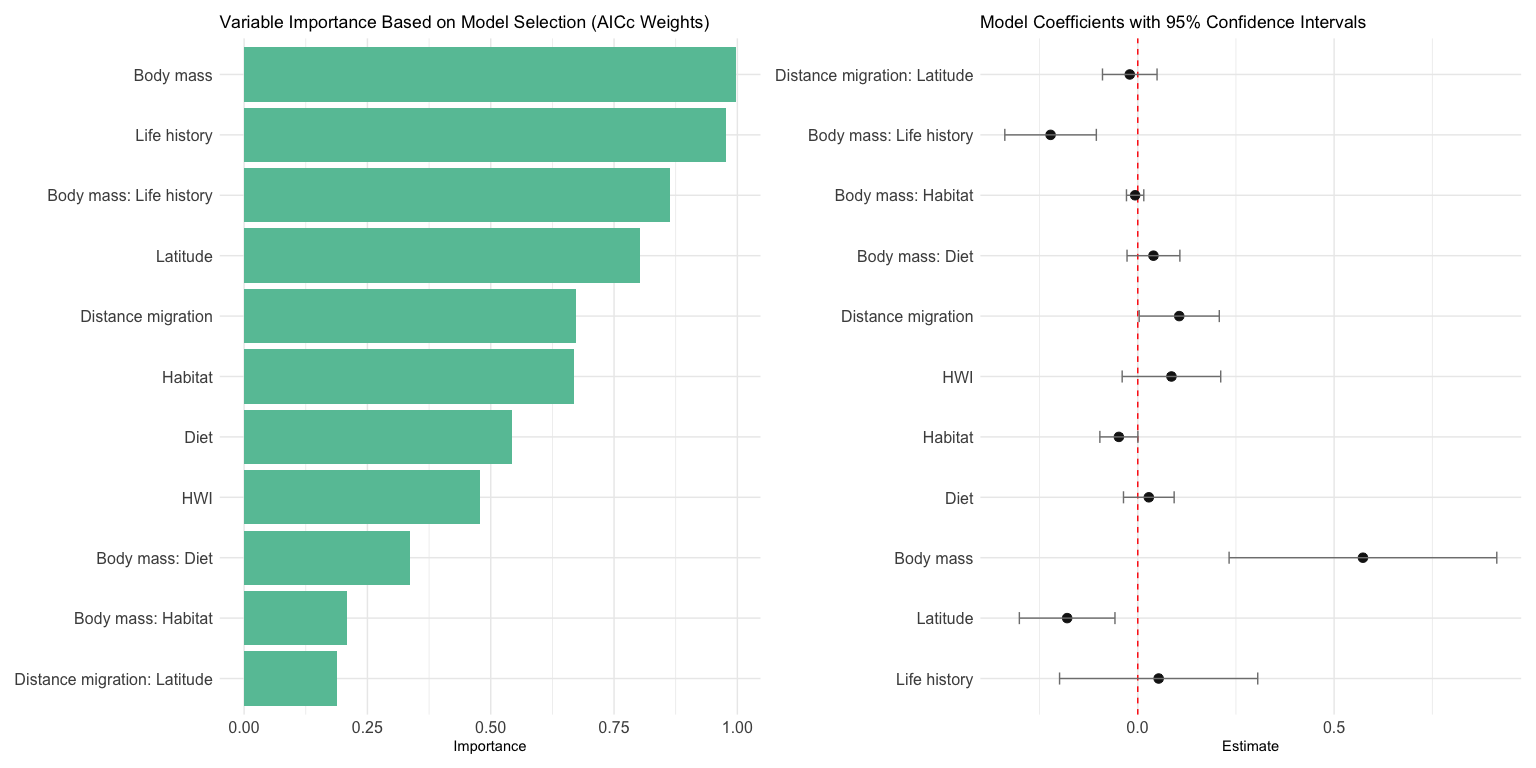


Supplementary Figure 3. Phylogenetic generalized least-squares model results for total dispersal. (a) Variable importance based on model selection and averaging, with values representing the summed Akaike weights across all models including each predictor. (b) Standardized coefficients and 95% confidence intervals for the predictors of total dispersal distance. Only variables included in the top-ranked models contributing to 95% of cumulative Akaike weight are shown. n = 138 species. Error bars represent 95% confidence intervals.

##

## **Supplementary Note 3: Phylogenetic generalized linear mixed models without interactions**


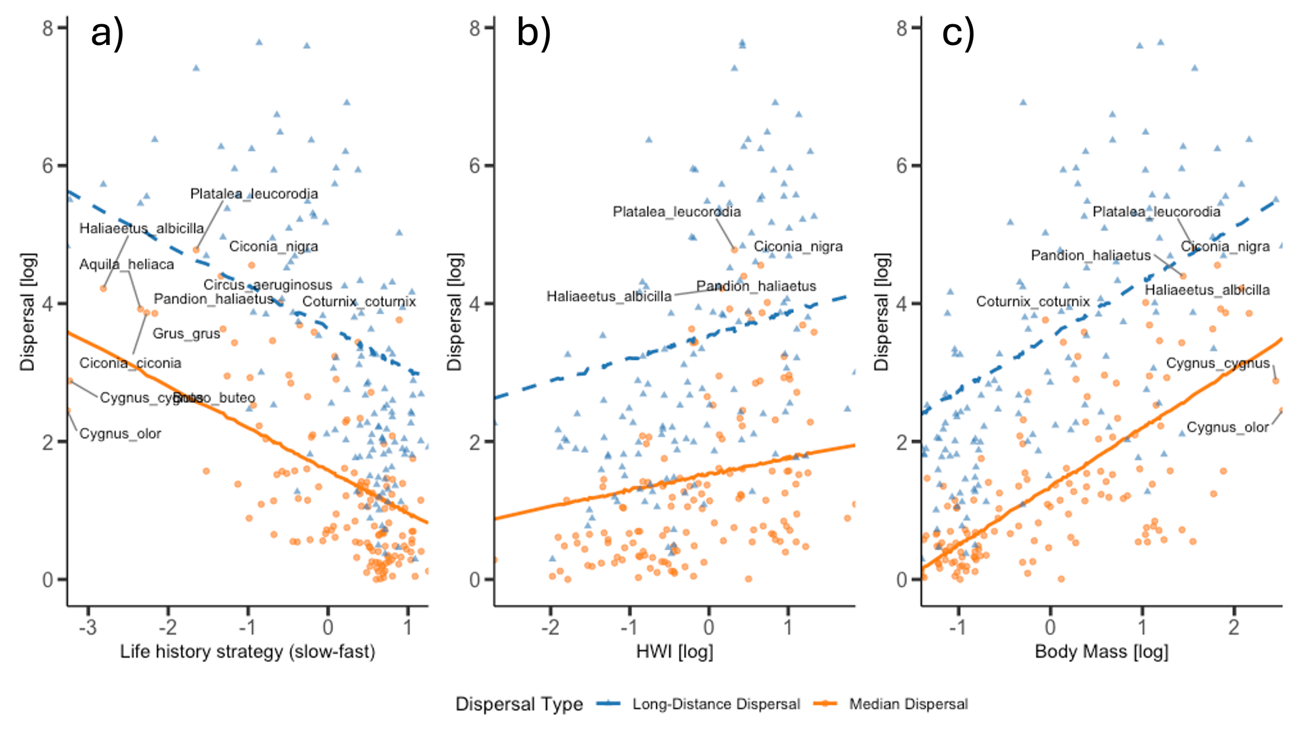


**Supplementary Figure 4.** Univariate trait–dispersal relationships for total dispersal. Panels show how median (orange circles) and long-distance (blue circles) dispersal among European birds (n = 138 species) varies with (a) life-history pace (slow–fast continuum), (b) body mass (log-transformed), and (c) Hand-Wing Index (log-transformed). Lines correspond to univariate phylogenetic generalized linear mixed models; solid lines indicate slopes significantly different from zero, dashed lines indicate non-significant relationships. Species names are labelled for selected data points. Dispersal estimates stem from the Weibull distribution^5^. Results from univariate models are provided in Supplementary Table 7

**
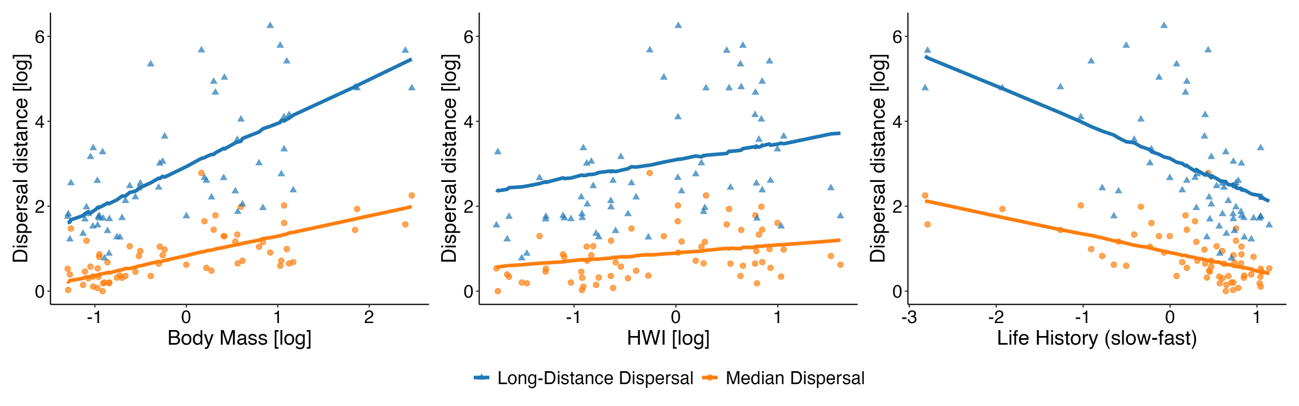
**

**Supplementary Figure 5.** Univariate trait–dispersal relationships for breeding dispersal. Panels show how median (orange circles) and long-distance (blue circles) breeding dispersal among European birds (n = 63 species) varies with (a) body mass (log-transformed), (b) Hand-Wing Index (log-transformed), and (c) life-history pace (slow–fast continuum). Lines correspond to univariate phylogenetic generalized linear mixed models; solid lines indicate slopes significantly different from zero, dashed lines indicate non-significant relationships. Dispersal estimates stem from the Weibull distribution^5^. Results from univariate models are provided in Supplementary Table 7.

**
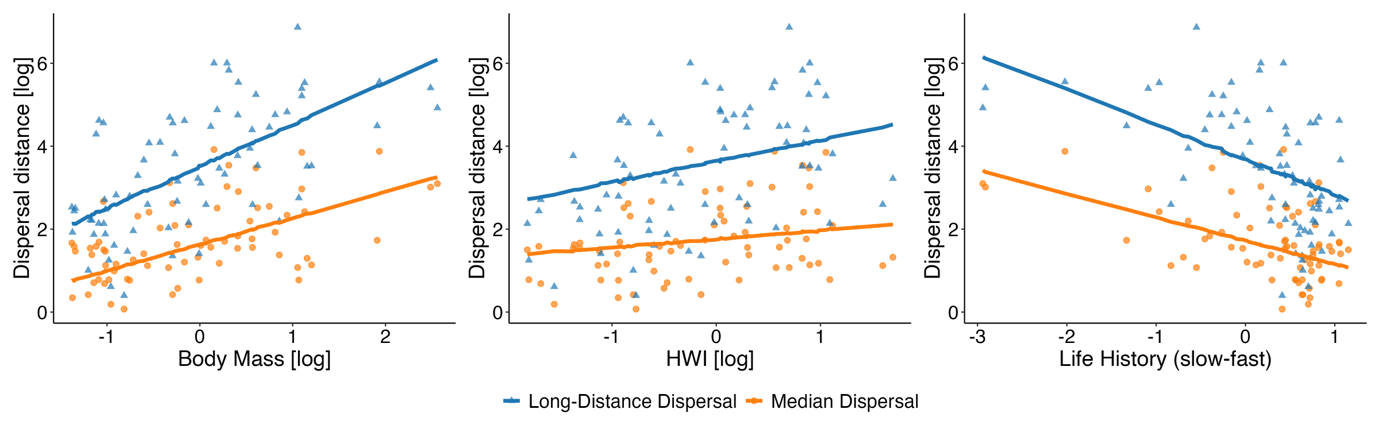
**

**Supplementary Figure 6.** Univariate trait–dispersal relationships for natal dispersal. Panels show how median (orange circles) and long-distance (blue circles) natal dispersal among European birds (n = 72 species) varies with (a) body mass (log-transformed), (b) Hand-Wing Index (log-transformed), and (c) life-history pace (slow–fast continuum). Lines correspond to univariate phylogenetic generalized linear mixed models; solid lines indicate slopes significantly different from zero, dashed lines indicate non-significant relationships. Dispersal estimates stem from the Weibull distribution ^5^. Results from univariate models are provided in Supplementary Table 7.

**
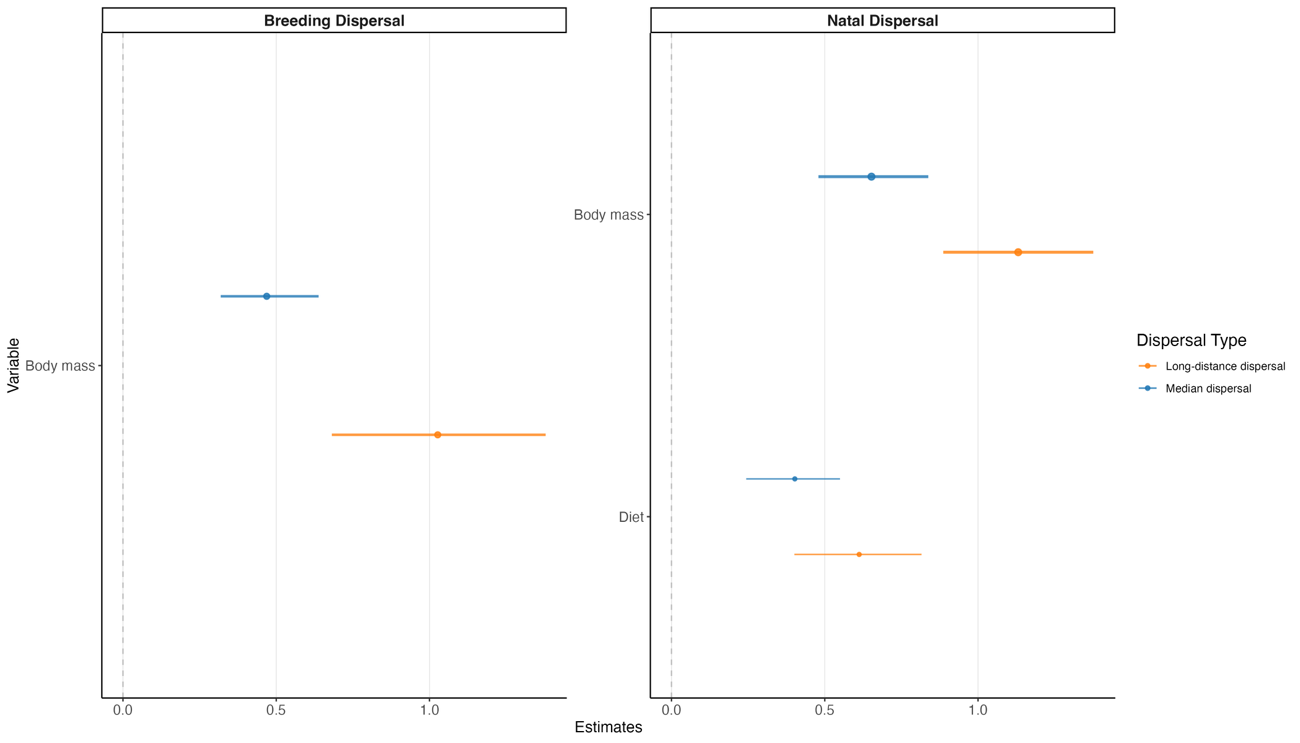
**

**Supplementary Figure 7.** Dispersal syndromes for breeding and natal dispersal. Standardized coefficients and 95% credible intervals of predictors of median and long-distance dispersal for (a) breeding dispersal (n = 63 species) and (b) natal dispersal (n = 72 species) among European birds based on phylogenetic generalized linear mixed models. Coefficients derive from reduced multivariate models with variables retained through projection predictive selection based on expected log predictive density. Variable importance is indicated by point size based on variable selection ranking. Dispersal estimates stem from the Weibull distribution^5^. Error bars represent 95% credible intervals from posterior distributions.

## **Supplementary Note 4: Interaction models**

Dispersal is a multicausal process in which the effects of individual traits may depend on the broader biological and environmental context rather than acting independently. We therefore tested a limited set of biologically motivated interaction terms, selected a priori based on theoretical expectations and empirical precedent, and constrained by sample size limitations that precluded exhaustive combinatorial testing.

**Rationale for selected interactions**

Body mass × life history. Allometric scaling governs key energetic and demographic trade-offs that jointly influence dispersal capacity. The relationship between life-history pace and dispersal may differ across body sizes because larger species face distinct constraints on survival, fecundity, and competitive ability compared to smaller species operating along the same slow–fast continuum^10^.

Body mass × diet. Trophic strategy determines spatial resource requirements and foraging behavior, which may modulate size-dependent dispersal patterns. Carnivorous species typically range over larger areas and may exhibit amplified body-size effects on dispersal relative to herbivores or omnivores^11,12^

Body mass × habitat openness. Habitat structure mediates movement costs through effects on flight efficiency, predation risk, and landscape permeability. The influence of body size on dispersal may therefore vary between open and forested environments, where these constraints differ substantially^12,13^

Migration distance × latitude. Latitudinal gradients in seasonality and environmental predictability shape both migratory behavior and dispersal decisions. We included this interaction to test whether the association between migration distance and dispersal propensity varies with breeding latitude, potentially reflecting geographic differences in spatial constraints and environmental stability ^14,15^

**Summary**

Interaction models occasionally improved explanatory power but did not consistently enhance predictive performance across dispersal types. Moreover, their inclusion reduced model comparability and interpretability. Given our primary objective of identifying general, transferable predictors of dispersal, we focused the main text on main-effect models to ensure transparency and reproducibility. Interaction results are presented here for completeness (Supplementary Figures 8–12).

**Supplementary Figure 8. Dispersal syndromes including interaction effects for breeding and natal dispersal.** Standardized coefficients and 95% credible intervals of predictors (main effects and interactions) for (a) breeding dispersal (n = 63 species) and (b) natal dispersal (n = 72 species), shown separately for median (orange) and long-distance (blue) dispersal. Models were fitted using phylogenetic generalized linear mixed models accounting for shared ancestry. Interaction terms include body mass × life history, body mass × diet, and body mass × habitat openness. Error bars represent 95% credible intervals from posterior distributions.

**Supplementary Figure 9. Dispersal syndromes including interaction effects for passerines.** Standardized coefficients and 95% credible intervals of predictors (main effects and interactions) for (a) long-distance and (b) median dispersal across total, breeding, and natal dispersal types within passerines (n = 68 species). Models were fitted with phylogenetic structure to control for evolutionary relationships. This sensitivity analysis tests whether trait–dispersal associations are consistent within a phylogenetically constrained subset. Error bars represent 95% credible intervals from posterior distributions.


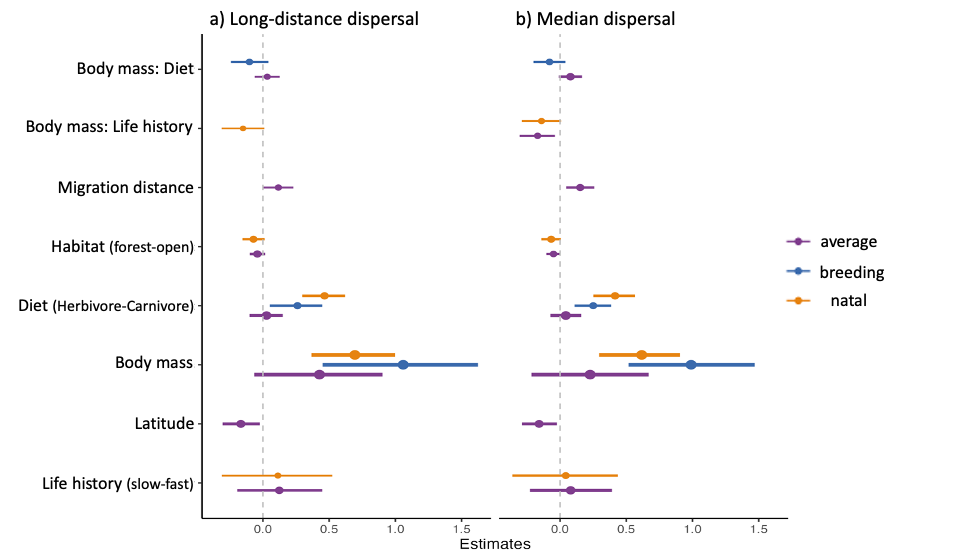
 **Supplementary Figure 10. Comparison of interaction models across dispersal types using Half-Cauchy kernels.** Standardized coefficients and 95% credible intervals for predictors and their interactions across (a) long-distance and (b) median dispersal in total (n = 138), breeding (n = 63), and natal (n = 72) datasets. Variable importance based on expected log predictive density is indicated by point size. Models were fitted using phylogenetic generalized linear mixed models with dispersal estimates derived from the Half-Cauchy distribution^5^. Error bars represent 95% credible intervals from posterior distributions.


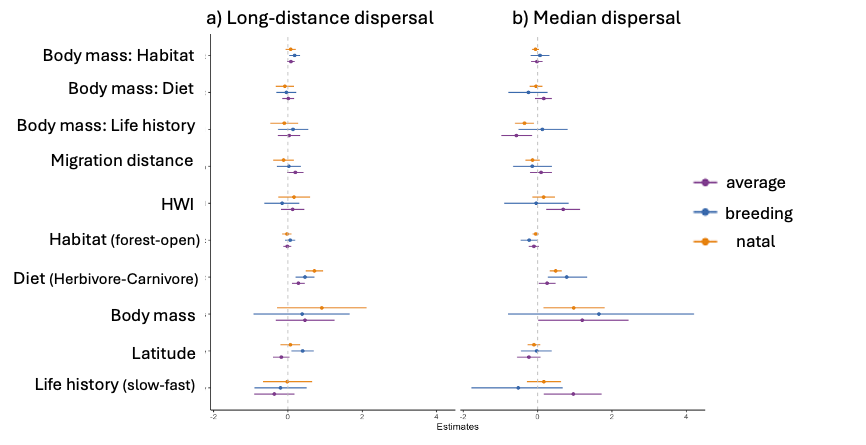


**Supplementary Figure 11. Full multivariate interaction model of dispersal syndromes.** Standardized coefficients and 95% credible intervals of all predictors (main effects and interactions) for (a) long-distance and (b) median dispersal across total (n = 138), breeding (n = 63), and natal (n = 72) datasets. Models include all covariates and interaction terms simultaneously and account for phylogenetic relatedness. This comprehensive model provides a benchmark against the simplified main-effect models presented in the main text. Error bars represent 95% credible intervals from posterior distributions.


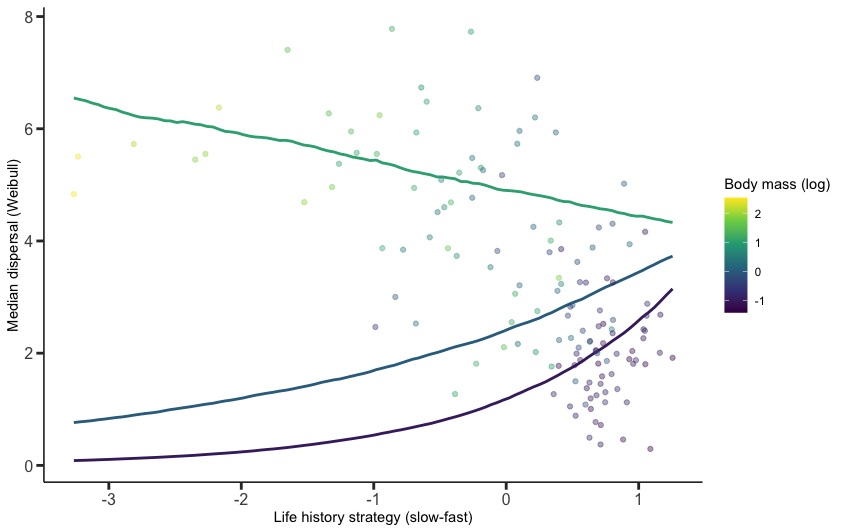


**Supplementary Figure 12**. **Two-way interaction between life history and body mass on median dispersal distances.** Predicted median dispersal distances across the life-history continuum as a function of body mass, based on phylogenetic generalized linear mixed models (n = 138 species). Lines represent marginal effects of the body mass × life history interaction at different body mass values (light to dark shading: small to large body mass). The interaction reveals that the relationship between life-history pace and dispersal depends on body size: larger, fast-paced species disperse farther than similarly sized slow-paced species. Dispersal estimates stem from the Weibull distribution.

## **Supplementary Note 5: Sensitivity analyses**

We tested the predictive performance of dispersal syndromes within and between bird orders, comparing our variable-selected multi-trait models and single-trait models with a model only calibrated with the phylogeny and a random null model (only intercept; Fig S3). This way, we could ascertain how robust our predictions of dispersal distances are across the bird phylogenetic tree. We selected four bird orders with a reasonable number of species to test between and within-order predictive performance (Accipitriformes 11 species, Anseriformes 12 species, Charadriiformes 11 species and Passeriformes 68 species).

Within-order predictive performance was assessed using five-fold cross-validation where species were partitioned into five folds, the multi-trait or single-trait models retrained on four folds and predicted to the hold-out fold of species (Fig S3). Between-order predictive performance was assessed by training the multi-trait and single-trait models on each of the four bird orders and then predicted dispersal distances to the remaining (Fig S3). We included the covariance matrix containing phylogenetic distances among species in all calibrated models, except for the random null model. In the test, we allowed the predictions the possibility of including new levels on this covariance matrix, meaning that the prediction will use the unconditional values for data with previously unobserved levels. To examine the predictive power of the single-trait, multi-trait models, only phylogenetic model, and random null model, and the ability of models to predict dispersal distances between and within orders correctly, we used the function ‘model_perfomance’ from performance R package ^16^. We used the r-squared value to evaluate the predictive performance.

**Supplementary Figure 13.** Predictive accuracy for breeding and natal dispersal. Predictive performance (R²) from within-order (orange) and between-order (teal) cross-validation for single-trait models and the multi-trait model (dispersal syndrome) for median dispersal distance in (a) breeding dispersal (n = 63 species) and (b) natal dispersal (n = 72 species). Point shapes indicate the order used as training data (squares: Accipitriformes; triangles: Anseriformes; crosses: Charadriiformes; asterisks: Passeriformes). Circles represent mean R² values; error bars represent ±1 standard deviation across cross-validation folds.


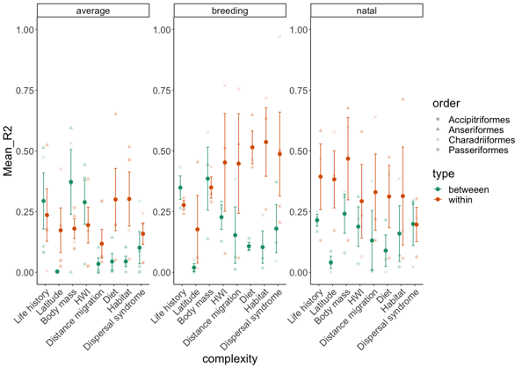


**Supplementary Figure 14.** Predictive accuracy for long-distance dispersal. Predictive performance (R²) from within-order (orange) and between-order (teal) cross-validation for single-trait models and the multi-trait model (dispersal syndrome) for long-distance dispersal in (a) total dispersal (n = 138 species), (b) natal dispersal (n = 72 species), and (c) breeding dispersal (n = 63 species). Point shapes indicate the order used as training data (squares: Accipitriformes; triangles: Anseriformes; crosses: Charadriiformes; asterisks: Passeriformes). Circles represent mean R² values; error bars represent ±1 standard deviation across cross-validation folds.

 **Supplementary Figure 15**. Comparison with null and phylogeny-only models. Predictive performance (R²) from within-order (orange) and between-order (teal) cross-validation comparing single-trait models, the multi-trait dispersal syndrome, a phylogeny-only model (fitted with only the phylogenetic covariance matrix), and a random null model (intercept only) for median total dispersal (n = 138 species). Point shapes indicate the order used as training data (squares: Accipitriformes; triangles: Anseriformes; crosses: Charadriiformes; asterisks: Passeriformes). Random model mean R²: within = 0.234 (standard deviation = 0.113), between = 0.030 (standard deviation = 0.046). Phylogenetic model mean R²: within = 0.241 (standard deviation = 0.209), between = 0.007 (standard deviation = 0.005). Circles represent mean R² values; error bars represent ±1 standard deviation across cross-validation folds.


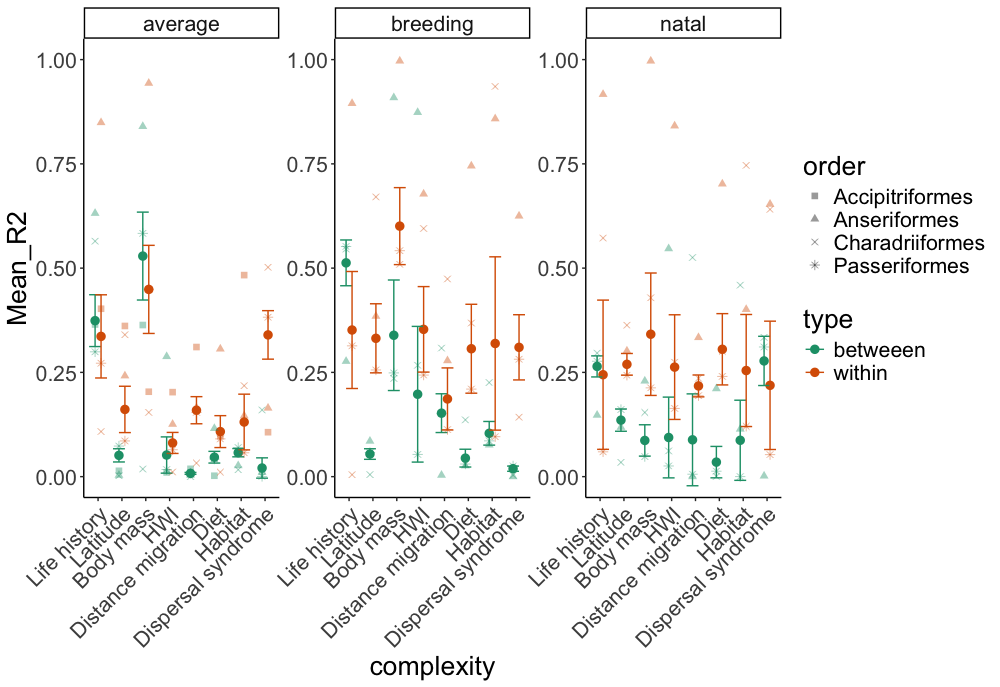


**Supplementary Figure 16.** Weighted predictive accuracy across orders. Predictive performance (weighted R², proportional to the number of species per order) from within-order (orange) and between-order (teal) cross-validation for single-trait models and the multi-trait model (dispersal syndrome) for median dispersal in (a) total dispersal (n = 138 species), (b) natal dispersal (n = 72 species), and (c) breeding dispersal (n = 63 species). Point shapes indicate the order used as training data (squares: Accipitriformes; triangles: Anseriformes; crosses: Charadriiformes; asterisks: Passeriformes). Weighting accounts for the contribution of larger orders to overall model performance. Circles represent mean R² values; error bars represent ±1 standard deviation across cross-validation folds.

## **Supplementary Tables**

**Supplementary Table 1.** Characterization of the traits considered for analyzing European bird dispersal syndromes.

| **Trait ID** | | **Trait name** | **Description** |  | **References** | |
| --- | --- | --- | --- | --- | --- | --- |
| a | Body mass | body weight (mean for male and female in breeding season) | | Storchova and Horak (2018) |  |  |
| b | Hand Wing Index [HWI] | Kipp’s distance corrected for wing size | | Sheard et al. 2020 |  |  |
| c | Diet | diet niche position along the gradient from species feeding obligatory on plants (1) to species feeding obligatory on animals (5) | | Reif et al. 2016 |  |  |
| d | Life history strategy | position along the slow-fast life history gradient revealed by a principal component analysis on six species' life-history traits | | Storchova and Horak (2018); Hanzelka et al. 2019 |  |  |
| e | Habitat preference | mean of species' positions along the gradient from closed forest (1) to open treeless habitat (7) | | Koschova et al. 2018 |  |  |
| f | Latitude | latitude of the center of species' breeding range in Europe (in decimal degrees) | | Koschova et al. 2019 |  |  |
| g | Migration distance | migration distance as a Great circle distance between centroids of species' breeding and non‐breeding ranges | | Hanzelka et al. 2019 |  |  |

**Supplementary Table 2.** Bayesian phylogenetic mixed models comparison using Leave-One-Out Cross-Validation (LOO-CV). Models include univariate (single-trait), multivariate reduced (selected through projection predictive feature selection), and multivariate complete (full model with all main effects but no interactions). elpd_loo represents the expected log predictive density, with higher values indicating better predictive performance. looic is the LOO Information Criterion (lower values indicate better fit). se_looic represents the standard error of looic. p_loo is the effective number of parameters, and n_params shows the actual number of model parameters. delta_looic and delta_elpd show differences relative to the best model (set to 0 for the top-performing model). The multivariate reduced model represents the optimal subset of predictors identified through Bayesian variable selection, delivering the smallest number of traits that achieve predictive performance similar to the full model. All models account for phylogenetic relatedness through random effects but include no trait interaction

| model_type | dispersal_type | distance_type | variable | elpd_loo | looic | se_looic | p_loo | n_params | delta_looic | delta_elpd |
| --- | --- | --- | --- | --- | --- | --- | --- | --- | --- | --- |
| univariate | total | long | log_HWI | -206,09 | 412,17 | 13,94 | 38,57 | 3 | 0 | 0 |
| univariate | total | long | diet | -206,61 | 413,22 | 14,41 | 41,63 | 3 | 1,05 | -0,52 |
| univariate | total | long | body_mass | -207,47 | 414,94 | 15,78 | 30,83 | 3 | 2,77 | -1,38 |
| univariate | total | long | PC1 | -207,75 | 415,51 | 15,01 | 31,7 | 3 | 3,34 | -1,66 |
| univariate | total | long | habita_for | -207,77 | 415,54 | 14,34 | 39,62 | 3 | 3,37 | -1,68 |
| univariate | total | long | Latitude | -208,15 | 416,29 | 14,83 | 41,11 | 3 | 4,12 | -2,06 |
| univariate | total | long | distance_mig | -208,18 | 416,37 | 14,65 | 39,99 | 3 | 4,2 | -2,09 |
| multivariate_reduced | total | long | reduced_model | -212,59 | 425,18 | 15,75 | 31,64 | 7 | 13,01 | -6,5 |
| multivariate_complete | total | long | full_model | -214,48 | 428,97 | 16,06 | 31,88 | 8 | 16,8 | -8,39 |
| multivariate_reduced | total | median | reduced_model | -149,69 | 299,38 | 16,37 | 16,24 | 6 | 0 | 0 |
| univariate | total | median | PC1 | -158,59 | 317,19 | 25,72 | 32,72 | 3 | 17,81 | -8,9 |
| univariate | total | median | body_mass | -158,77 | 317,54 | 32,94 | 32,68 | 3 | 18,16 | -9,08 |
| multivariate_complete | total | median | full_model | -162,18 | 324,36 | 30,52 | 27,98 | 8 | 24,98 | -12,49 |
| univariate | total | median | log_HWI | -167,54 | 335,07 | 20,27 | 35,36 | 3 | 35,69 | -17,85 |
| univariate | total | median | habita_for | -168,68 | 337,37 | 20,64 | 37,5 | 3 | 37,99 | -18,99 |
| univariate | total | median | distance_mig | -168,96 | 337,93 | 20,35 | 36,66 | 3 | 38,55 | -19,27 |
| univariate | total | median | diet | -169,46 | 338,93 | 20,91 | 38,05 | 3 | 39,55 | -19,77 |
| univariate | total | median | Latitude | -170,49 | 340,98 | 21,09 | 36,67 | 3 | 41,6 | -20,8 |
| multivariate_reduced | breeding | long | reduced_model | -93,36 | 186,72 | 12,51 | 8,67 | 2 | 0 | 0 |
| multivariate_complete | breeding | long | full_model | -95,89 | 191,79 | 13,7 | 11,73 | 8 | 5,07 | -2,53 |
| univariate | breeding | long | log_HWI | -206,09 | 412,17 | 13,94 | 38,57 | 3 | 225,45 | -112,73 |
| univariate | breeding | long | diet | -206,61 | 413,22 | 14,41 | 41,63 | 3 | 226,5 | -113,25 |
| univariate | breeding | long | body_mass | -207,47 | 414,94 | 15,78 | 30,83 | 3 | 228,22 | -114,11 |
| univariate | breeding | long | PC1 | -207,75 | 415,51 | 15,01 | 31,7 | 3 | 228,79 | -114,39 |
| univariate | breeding | long | habita_for | -207,77 | 415,54 | 14,34 | 39,62 | 3 | 228,82 | -114,41 |
| univariate | breeding | long | Latitude | -208,15 | 416,29 | 14,83 | 41,11 | 3 | 229,57 | -114,79 |
| univariate | breeding | long | distance_mig | -208,18 | 416,37 | 14,65 | 39,99 | 3 | 229,65 | -114,82 |
| multivariate_reduced | breeding | median | reduced_model | -42,76 | 85,52 | 17,37 | 8,81 | 2 | 0 | 0 |
| multivariate_complete | breeding | median | full_model | -45,28 | 90,57 | 19,66 | 11,73 | 8 | 5,05 | -2,52 |
| univariate | breeding | median | PC1 | -158,59 | 317,19 | 25,72 | 32,72 | 3 | 231,67 | -115,83 |
| univariate | breeding | median | body_mass | -158,77 | 317,54 | 32,94 | 32,68 | 3 | 232,02 | -116,01 |
| univariate | breeding | median | log_HWI | -167,54 | 335,07 | 20,27 | 35,36 | 3 | 249,55 | -124,78 |
| univariate | breeding | median | habita_for | -168,68 | 337,37 | 20,64 | 37,5 | 3 | 251,85 | -125,92 |
| univariate | breeding | median | distance_mig | -168,96 | 337,93 | 20,35 | 36,66 | 3 | 252,41 | -126,2 |
| univariate | breeding | median | diet | -169,46 | 338,93 | 20,91 | 38,05 | 3 | 253,41 | -126,7 |
| univariate | breeding | median | Latitude | -170,49 | 340,98 | 21,09 | 36,67 | 3 | 255,46 | -127,73 |
| multivariate_reduced | natal | long | reduced_model | -103,61 | 207,22 | 12,62 | 5,24 | 3 | 0 | 0 |
| multivariate_complete | natal | long | full_model | -107,75 | 215,5 | 13,48 | 10,26 | 8 | 8,28 | -4,14 |
| univariate | natal | long | log_HWI | -206,09 | 412,17 | 13,94 | 38,57 | 3 | 204,95 | -102,48 |
| univariate | natal | long | diet | -206,61 | 413,22 | 14,41 | 41,63 | 3 | 206 | -103 |
| univariate | natal | long | body_mass | -207,47 | 414,94 | 15,78 | 30,83 | 3 | 207,72 | -103,86 |
| univariate | natal | long | PC1 | -207,75 | 415,51 | 15,01 | 31,7 | 3 | 208,29 | -104,14 |
| univariate | natal | long | habita_for | -207,77 | 415,54 | 14,34 | 39,62 | 3 | 208,32 | -104,16 |
| univariate | natal | long | Latitude | -208,15 | 416,29 | 14,83 | 41,11 | 3 | 209,07 | -104,54 |
| univariate | natal | long | distance_mig | -208,18 | 416,37 | 14,65 | 39,99 | 3 | 209,15 | -104,57 |
| multivariate_reduced | natal | median | reduced_model | -75,3 | 150,6 | 9,99 | 6,87 | 3 | 0 | 0 |
| multivariate_complete | natal | median | full_model | -76,77 | 153,54 | 9,78 | 11,62 | 8 | 2,94 | -1,47 |
| univariate | natal | median | PC1 | -158,59 | 317,19 | 25,72 | 32,72 | 3 | 166,59 | -83,29 |
| univariate | natal | median | body_mass | -158,77 | 317,54 | 32,94 | 32,68 | 3 | 166,94 | -83,47 |
| univariate | natal | median | log_HWI | -167,54 | 335,07 | 20,27 | 35,36 | 3 | 184,47 | -92,24 |
| univariate | natal | median | habita_for | -168,68 | 337,37 | 20,64 | 37,5 | 3 | 186,77 | -93,38 |
| univariate | natal | median | distance_mig | -168,96 | 337,93 | 20,35 | 36,66 | 3 | 187,33 | -93,66 |
| univariate | natal | median | diet | -169,46 | 338,93 | 20,91 | 38,05 | 3 | 188,33 | -94,16 |
| univariate | natal | median | Latitude | -170,49 | 340,98 | 21,09 | 36,67 | 3 | 190,38 | -95,19 |

**Supplementary Table 3.** Summary of optimal Bayesian phylogenetic mixed models selected through projection predictive feature selection. Models include no trait interactions and account for phylogenetic relatedness. Age Class refers to the dispersal life stage: total (pooling all age classes), breeding (between subsequent breeding attempts), and natal (from natal site to first breeding site). Dispersal Type indicates whether the model predicts median dispersal distances or long-distance dispersal (95th percentile). N Predictors shows the number of traits retained in the reduced model after variable selection. R² (Marg. / Cond.) represents marginal R² (variance explained by fixed effects only) and conditional R² (variance explained by both fixed and random effects). Phylogenetic Signal (λ) indicates the strength of phylogenetic correlation in dispersal traits, with values near 0 suggesting trait independence from phylogeny and values near 1 indicating strong phylogenetic constraint; 95% credible intervals are shown in brackets. Phylogenetic signal was estimated from the models following P. Bürkner's recommendations (https://cran.r-project.org/web/packages/brms/vignettes/brms_phylogenetics.html), using the 'hypothesis' method and substituting π²/3 for the residual variance. Stacking refers to model averaging of multiple competing models, with uncertainty percentage indicating the proportion of posterior samples where model weights were uncertain. All models were selected based on optimal predictive performance using Leave-One-Out Cross-Validation.

| Model | Age Class | Dispersal Type | N Predictors | R^2^ (Marg. / Cond.) | Phylogenetic Signal |
| --- | --- | --- | --- | --- | --- |
| Total Median (Reduced) | total | Median dispersal | 5 | 0.522 / 0.668 | 0.548 [0.115, 0.856] |
| Total Long (Stacking) [2 models] | total | Long-distance dispersal | 2 | 0.513 / 0.641 (35.9% unc.) | 0.895 [0.795, 0.995] |
| Breeding Median (Reduced) | breeding | Median dispersal | 1 | 0.476 / 0.493 | 0.272 [0.007, 0.67] |
| Breeding Long (Reduced) | breeding | Long-distance dispersal | 1 | 0.479 / 0.528 | 0.266 [0.003, 0.665] |
| Natal Median (Reduced) | natal | Median dispersal | 2 | 0.506 / 0.525 | 0.139 [0, 0.551] |
| Natal Long (Reduced) | natal | Long-distance dispersal | 2 | 0.57 / 0.58 | 0.083 [0, 0.377] |

**Supplementary Table 4.** Standardized coefficients from optimal Bayesian phylogenetic mixed models for dispersal traits. Coefficients represent the effect size of each predictor on log-transformed dispersal distances, derived from models selected through projection predictive feature selection. Total dispersal (pooling all age classes) for median/long-distance dispersal; Breeding dispersal for median/long-distance dispersal; natal dispersal for median/long-distance dispersal. All models account for phylogenetic relatedness and include no trait interactions. Body mass and HWI (Hand Wing Index) were log-transformed prior to standardization. Diet represents the herbivore-carnivore gradient (1=obligate plant feeders, 5=obligate animal feeders). Habitat indicates landscape openness preference (1=forest interior, 7=open treeless landscape). Life history represents the slow-fast continuum (negative values=slow strategies, positive values=fast strategies). Latitude is the mean breeding latitude. Dashes (—) indicate variables not retained in the reduced model. Statistical significance: *** p < 0.001. All continuous variables were standardized (mean=0, SD=1) before analysis.

| Variable | Total - Median | Total - Long-distance | Breeding - Median | Breeding - Long-distance | Natal - Median | Natal - Long-distance |
| --- | --- | --- | --- | --- | --- | --- |
| Body mass | 0.504*** | - | 0.469*** | 1.027*** | 0.652*** | 1.131*** |
| Diet | 0.061 | -0.041 | - | - | 0.402*** | 0.612*** |
| HWI | - | 0.215*** | - | - | - | - |
| Habitat | -0.056 | - | - | - | - | - |
| Latitude | -0.193*** | - | - | - | - | - |
| Life history | -0.383*** | - | - | - | - | - |

**Supplementary Table 5.** Predictive accuracy of single-trait and multi-trait models to estimate median dispersal for missing species within and between orders for natal, breeding, and total dispersal. For the within-order cross-validation, we used a five-fold design refitting the trait models to 80% of the species and cross-predicting to the hold-out 20% of the species. For the between-order cross-validation, we used a four-fold design and selected four orders with a reasonable number of species (Accipitriformes 11 species, Anseriformes 12 species, Charadriiformes 11 species and Passeriformes 68 species), refitted the trait models with one order, and cross-predicted to the other three orders to assess prediction accuracy. sd (standard deviation). se (standard error).

| Dispersal type | Prediction | Complexity | Mean R^2^ | sd | se |
| --- | --- | --- | --- | --- | --- |
| total | between | Life history | 0,465 | 0,158 | 0,079 |
| total | between | Latitude | 0,023 | 0,034 | 0,017 |
| total | between | Body mass | 0,392 | 0,260 | 0,130 |
| total | between | HWI | 0,211 | 0,061 | 0,031 |
| total | between | Distance migration | 0,008 | 0,008 | 0,004 |
| total | between | Diet | 0,051 | 0,047 | 0,024 |
| total | between | Habitat | 0,042 | 0,025 | 0,012 |
| total | between | Dispersal syndrome | 0,104 | 0,099 | 0,049 |
| total | within | Life history | 0,408 | 0,318 | 0,159 |
| total | within | Latitude | 0,257 | 0,126 | 0,063 |
| total | within | Body mass | 0,454 | 0,265 | 0,133 |
| total | within | HWI | 0,256 | 0,214 | 0,107 |
| total | within | Distance migration | 0,166 | 0,114 | 0,057 |
| total | within | Diet | 0,126 | 0,126 | 0,063 |
| total | within | Habitat | 0,226 | 0,184 | 0,092 |
| total | within | Dispersal syndrome | 0,263 | 0,245 | 0,123 |
| breeding | between | Life history | 0,458 | 0,157 | 0,091 |
| breeding | between | Latitude | 0,049 | 0,041 | 0,024 |
| breeding | between | Body mass | 0,405 | 0,076 | 0,044 |
| breeding | between | HWI | 0,219 | 0,143 | 0,082 |
| breeding | between | Distance migration | 0,154 | 0,152 | 0,088 |
| breeding | between | Diet | 0,064 | 0,062 | 0,036 |
| breeding | between | Habitat | 0,129 | 0,084 | 0,048 |
| breeding | between | Dispersal syndrome | 0,305 | 0,212 | 0,122 |
| breeding | within | Life history | 0,404 | 0,452 | 0,261 |
| breeding | within | Latitude | 0,437 | 0,212 | 0,123 |
| breeding | within | Body mass | 0,638 | 0,357 | 0,206 |
| breeding | within | HWI | 0,329 | 0,268 | 0,155 |
| breeding | within | Distance migration | 0,288 | 0,181 | 0,104 |
| breeding | within | Diet | 0,441 | 0,275 | 0,159 |
| breeding | within | Habitat | 0,630 | 0,464 | 0,268 |
| breeding | within | Dispersal syndrome | 0,625 | 0,334 | 0,193 |
| natal | between | Life history | 0,240 | 0,081 | 0,047 |
| natal | between | Latitude | 0,105 | 0,064 | 0,037 |
| natal | between | Body mass | 0,144 | 0,091 | 0,053 |
| natal | between | HWI | 0,211 | 0,291 | 0,168 |
| natal | between | Distance migration | 0,177 | 0,302 | 0,174 |
| natal | between | Diet | 0,075 | 0,118 | 0,068 |
| natal | between | Habitat | 0,191 | 0,239 | 0,138 |
| natal | between | Dispersal syndrome | 0,197 | 0,179 | 0,103 |
| natal | within | Life history | 0,516 | 0,431 | 0,249 |
| natal | within | Latitude | 0,303 | 0,060 | 0,035 |
| natal | within | Body mass | 0,546 | 0,405 | 0,234 |
| natal | within | HWI | 0,426 | 0,363 | 0,210 |
| natal | within | Distance migration | 0,255 | 0,072 | 0,041 |
| natal | within | Diet | 0,414 | 0,251 | 0,145 |
| natal | within | Habitat | 0,423 | 0,313 | 0,181 |
| natal | within | Dispersal syndrome | 0,373 | 0,315 | 0,182 |

**Supplementary Table 6.** Predictive accuracy of single-trait and multi-trait models to estimate long dispersal for missing species within and between orders for natal, breeding, and total dispersal. For the within-order cross-validation, we used a five-fold design refitting the trait models to 80% of the species and cross-predicting to the hold-out 20% of the species. For the between-order cross-validation, we used a four-fold design and selected four orders with a reasonable number of species (Accipitriformes 11 species, Anseriformes 12 species, Charadriiformes 11 species and Passeriformes 68 species), refitted the trait models with one order, and cross-predicted to the other three orders to assess prediction accuracy. sd (standard deviation). se (standard error).

| Dispersal type | Prediction | Complexity | Mean R^2^ | sd | se | ci |
| --- | --- | --- | --- | --- | --- | --- |
| total | betweeen | Life history | 0,294 | 0,231 | 0,115 | 0,367 |
| total | betweeen | Latitude | 0,003 | 0,003 | 0,001 | 0,004 |
| total | betweeen | Body mass | 0,372 | 0,266 | 0,133 | 0,424 |
| total | betweeen | HWI | 0,289 | 0,181 | 0,091 | 0,288 |
| total | betweeen | Distance migration | 0,035 | 0,048 | 0,024 | 0,077 |
| total | betweeen | Diet | 0,044 | 0,067 | 0,033 | 0,106 |
| total | betweeen | Habitat | 0,045 | 0,043 | 0,022 | 0,069 |
| total | betweeen | Dispersal syndrome | 0,101 | 0,132 | 0,066 | 0,210 |
| total | within | Life history | 0,236 | 0,216 | 0,108 | 0,344 |
| total | within | Latitude | 0,173 | 0,184 | 0,092 | 0,293 |
| total | within | Body mass | 0,180 | 0,081 | 0,041 | 0,129 |
| total | within | HWI | 0,194 | 0,147 | 0,074 | 0,234 |
| total | within | Distance migration | 0,118 | 0,118 | 0,059 | 0,187 |
| total | within | Diet | 0,300 | 0,256 | 0,128 | 0,407 |
| total | within | Habitat | 0,302 | 0,223 | 0,112 | 0,355 |
| total | within | Dispersal syndrome | 0,159 | 0,087 | 0,044 | 0,139 |
| breeding | betweeen | Life history | 0,349 | 0,084 | 0,048 | 0,208 |
| breeding | betweeen | Latitude | 0,020 | 0,029 | 0,016 | 0,071 |
| breeding | betweeen | Body mass | 0,386 | 0,224 | 0,129 | 0,557 |
| breeding | betweeen | HWI | 0,227 | 0,087 | 0,050 | 0,216 |
| breeding | betweeen | Distance migration | 0,153 | 0,202 | 0,116 | 0,501 |
| breeding | betweeen | Diet | 0,108 | 0,028 | 0,016 | 0,070 |
| breeding | betweeen | Habitat | 0,104 | 0,116 | 0,067 | 0,287 |
| breeding | betweeen | Dispersal syndrome | 0,180 | 0,170 | 0,098 | 0,423 |
| breeding | within | Life history | 0,276 | 0,033 | 0,019 | 0,081 |
| breeding | within | Latitude | 0,177 | 0,241 | 0,139 | 0,599 |
| breeding | within | Body mass | 0,349 | 0,076 | 0,044 | 0,189 |
| breeding | within | HWI | 0,452 | 0,349 | 0,202 | 0,867 |
| breeding | within | Distance migration | 0,448 | 0,355 | 0,205 | 0,882 |
| breeding | within | Diet | 0,515 | 0,117 | 0,067 | 0,290 |
| breeding | within | Habitat | 0,537 | 0,244 | 0,141 | 0,606 |
| breeding | within | Dispersal syndrome | 0,487 | 0,346 | 0,173 | 0,550 |
| natal | betweeen | Life history | 0,214 | 0,043 | 0,025 | 0,106 |
| natal | betweeen | Latitude | 0,041 | 0,043 | 0,025 | 0,108 |
| natal | betweeen | Body mass | 0,241 | 0,140 | 0,081 | 0,347 |
| natal | betweeen | HWI | 0,188 | 0,141 | 0,081 | 0,350 |
| natal | betweeen | Distance migration | 0,131 | 0,213 | 0,123 | 0,530 |
| natal | betweeen | Diet | 0,089 | 0,112 | 0,065 | 0,278 |
| natal | betweeen | Habitat | 0,159 | 0,198 | 0,114 | 0,492 |
| natal | betweeen | Dispersal syndrome | 0,199 | 0,154 | 0,089 | 0,381 |
| natal | within | Life history | 0,394 | 0,234 | 0,135 | 0,582 |
| natal | within | Latitude | 0,383 | 0,204 | 0,118 | 0,507 |
| natal | within | Body mass | 0,468 | 0,294 | 0,170 | 0,730 |
| natal | within | HWI | 0,293 | 0,263 | 0,152 | 0,652 |
| natal | within | Distance migration | 0,330 | 0,272 | 0,157 | 0,675 |
| natal | within | Diet | 0,312 | 0,222 | 0,128 | 0,553 |
| natal | within | Habitat | 0,314 | 0,350 | 0,202 | 0,869 |
| natal | within | Dispersal syndrome | 0,197 | 0,122 | 0,071 | 0,303 |

**Supplementary Table 7:** Standardized coefficients from univariate Bayesian phylogenetic mixed models for dispersal traits. Each model includes a single predictor and accounts for phylogenetic relatedness. Values show posterior mean coefficient estimates with 95% credible intervals in parentheses, followed by conditional R² (variance explained by fixed and random effects) in brackets. Total refers to average dispersal pooling all age classes; Natal dispersal is from natal site to first breeding site; Breeding dispersal is between subsequent breeding attempts. Median and Long-distance refer to median dispersal distances and 95th percentile of dispersal kernels, respectively. Body mass and HWI (Hand Wing Index) were log-transformed prior to standardization. Diet represents the herbivore-carnivore gradient (1=obligate plant feeders, 5=obligate animal feeders). Habitat openness indicates landscape preference (1=forest interior, 7=open treeless landscape). Life history represents the slow-fast continuum. Latitude is mean breeding latitude. Migration distance is the great circle distance between breeding and non-breeding ranges. All continuous variables were standardized (mean=0, SD=1). Asterisks (*) indicate 95% credible intervals that do not overlap zero.

| Variable | Total - Median | Total - Long-distance | Natal - Median | Natal - Long-distance | Breeding - Median | Breeding - Long-distance |
| --- | --- | --- | --- | --- | --- | --- |
| Body mass | 0.845 (0.595, 1.096) [R² = 0.690]* | 0.786 (0.365, 1.194) [R² = 0.716]* | 0.636 (0.343, 0.947) [R² = 0.507]* | 1.008 (0.588, 1.426) [R² = 0.516]* | 0.468 (0.289, 0.661) [R² = 0.484]* | 1.020 (0.619, 1.442) [R² = 0.520]* |
| Diet | -0.014 (-0.221, 0.194) [R² = 0.657] | -0.115 (-0.416, 0.176) [R² = 0.751] | 0.286 (0.037, 0.525) [R² = 0.423]* | 0.457 (0.090, 0.833) [R² = 0.487]* | 0.056 (-0.108, 0.219) [R² = 0.340] | 0.173 (-0.210, 0.557) [R² = 0.493] |
| HWI | 0.240 (0.003, 0.477) [R² = 0.656]* | 0.335 (-0.001, 0.662) [R² = 0.745] | 0.206 (-0.115, 0.514) [R² = 0.403] | 0.511 (0.028, 0.982) [R² = 0.452]* | 0.183 (-0.043, 0.394) [R² = 0.332] | 0.399 (-0.094, 0.884) [R² = 0.467] |
| Habitat openness | -0.024 (-0.111, 0.061) [R² = 0.658] | 0.009 (-0.108, 0.126) [R² = 0.742] | -0.004 (-0.121, 0.112) [R² = 0.414] | 0.004 (-0.176, 0.182) [R² = 0.463] | 0.016 (-0.065, 0.098) [R² = 0.338] | 0.048 (-0.118, 0.216) [R² = 0.480] |
| Latitude | -0.058 (-0.269, 0.146) [R² = 0.646] | 0.030 (-0.235, 0.306) [R² = 0.744] | -0.212 (-0.441, 0.015) [R² = 0.440] | -0.100 (-0.432, 0.240) [R² = 0.464] | -0.113 (-0.280, 0.056) [R² = 0.353] | 0.050 (-0.307, 0.408) [R² = 0.487] |
| Life history | -0.614 (-0.813, -0.417) [R² = 0.689]* | -0.592 (-0.907, -0.291) [R² = 0.718]* | -0.567 (-0.842, -0.300) [R² = 0.536]* | -0.842 (-1.274, -0.421) [R² = 0.548]* | -0.431 (-0.603, -0.262) [R² = 0.507]* | -0.860 (-1.272, -0.450) [R² = 0.539]* |
| Migration distance | -0.017 (-0.181, 0.151) [R² = 0.652] | 0.004 (-0.219, 0.223) [R² = 0.741] | -0.089 (-0.290, 0.112) [R² = 0.424] | -0.082 (-0.410, 0.259) [R² = 0.463] | -0.040 (-0.187, 0.109) [R² = 0.343] | -0.026 (-0.349, 0.287) [R² = 0.491] |

## **Supplementary References**

1. Penone, C. *et al.* Imputation of missing data in life-history trait datasets: which approach performs the best? *Methods in Ecology and Evolution* **5**, 961–970 (2014).

2. Stekhoven, D. J. & Bühlmann, P. MissForest—non-parametric missing value imputation for mixed-type data. *Bioinformatics* **28**, 112–118 (2012).

3. Buuren, S. van & Groothuis-Oudshoorn, K. mice: Multivariate Imputation by Chained Equations in R. *Journal of Statistical Software* **45**, 1–67 (2011).

4. Bürkner, P.-C. brms: An R package for Bayesian multilevel models using Stan. *Journal of statistical software* **80**, 1–28 (2017).

5. Fandos, G. *et al.* Standardised empirical dispersal kernels emphasise the pervasiveness of long-distance dispersal in European birds. *Journal of Animal Ecology* **92**, 158–170 (2023).

6. Piironen, J., Paasiniemi, M., Catalina, A., Weber, F. & Vehtari, A. projpred: Projection Predictive Feature Selection. (2023).

7. Freckleton, R. P., Harvey, P. H. & Pagel, M. Phylogenetic analysis and comparative data: a test and review of evidence. *The American Naturalist* (2015).

8. Burnham, K. & Anderson, D. *Model Selection and Multi-Model Inference*. (Springer, New York, 2002).

9. Barton, K. & Barton, M. K. Package ‘mumin’. *Version* **1**, 18 (2015).

10. Sæther, B.-E. & Bakke, Ø. AVIAN LIFE HISTORY VARIATION AND CONTRIBUTION OF DEMOGRAPHIC TRAITS TO THE POPULATION GROWTH RATE. *Ecology* **81**, 642–653 (2000).

11. Sutherland, G. D., Harestad, A. S., Price, K. & Lertzman, K. P. Scaling of natal dispersal distances in terrestrial birds and mammals. *Ecology and Society* **4**, (2000).

12. Stevens, V. M. *et al.* A comparative analysis of dispersal syndromes in terrestrial and semi-terrestrial animals. *Ecol Lett* **17**, 1039–1052 (2014).

13. Ronce, O. How does it feel to be like a rolling stone? Ten questions about dispersal evolution. *Annu. Rev. Ecol. Evol. Syst.* **38**, 231–253 (2007).

14. Goossens, S., Wybouw, N., Van Leeuwen, T. & Bonte, D. The physiology of movement. *Mov Ecol* **8**, 5 (2020).

15. Ronce, O. & Clobert, J. Dispersal syndromes. *Dispersal ecology and evolution* **155**, 119–138 (2012).

16. Lüdecke, D., Ben-Shachar, M. S., Patil, I., Waggoner, P. & Makowski, D. performance: An R Package for Assessment, Comparison and Testing of Statistical Models. *Journal of Open Source Software* **6**, 3139 (2021).
